# Size spectra of the edaphic fauna of typical Argiudol soils of the Rolling Pampa Region, Argentina

**DOI:** 10.3897/BDJ.11.e113074

**Published:** 2023-12-18

**Authors:** Víctor N. Velazco, Rosana V Sandler, Maria Cynthia Valeria Sanabria, Liliana B Falco, Carlos E Coviella, Leonardo A. Saravia

**Affiliations:** 1 Instituto de Ecología y Desarrollo Sustentable (INEDES) - Dept. of Basic Sciences, Universidad Nacional de Luján, Luján, Argentina Instituto de Ecología y Desarrollo Sustentable (INEDES) - Dept. of Basic Sciences, Universidad Nacional de Luján Luján Argentina; 2 Centro Austral de Investigaciones Científicas (CADIC - CONICET), Ushuaia, Argentina Centro Austral de Investigaciones Científicas (CADIC - CONICET) Ushuaia Argentina

**Keywords:** Soil fauna, soil invertebrates, Acari, Collembola, earthworms, body mass, body length, body width, Rolling Pampas, morphological traits, intensities of land use, occurrence, specimen

## Abstract

**Background:**

Soil-dwelling organisms populate the spaces—referred to as interstices—between the litter on the soil surface and the pores in the soil's organo-mineral matrix. These organisms have pivotal roles in soil ecosystem functions, such as the breakdown and decomposition of organic matter, the dispersal of bacterial and fungal spores and biological habitat transformation. These functions, in turn, contribute to broader ecosystem services like carbon and nutrient cycling, soil organic matter regulation and both chemical and physical soil fertility.

This study provides morphological data pertaining to a range of soil organism sizes, specifically in Argiudol soils subjected to varying levels of agricultural activity in the Rolling Pampas Region, one of the world's most extensive and fertile plains.

The primary focus is on soil microarthropods—namely, Acari (mites) and Collembola (springtails)—with a body width of less than 2 mm. These organisms constitute the majority of life in the intricate soil pore network. Additionally, the study documents species of earthworms (Oligochaeta, Crassiclitelata), recognised as ecosystem engineers for their ability to create physical channels in the soil matrix and to distribute organic matter. Moreover, the study includes measurements of morphological traits of soil-dwelling "macrofauna" (organisms with a body width greater than 2 mm), which are also implicated in various soil ecosystem functions. These include population regulation by apex predators, organic matter decomposition, biogenic structure formation, nutrient mobilisation and herbivory.

**New information:**

In this paper, we report both the geographical locations and individual measurements of key morphological traits for over 7,000 specimens, covering a range of soil-dwelling organisms. These include springtails (Entognatha, Collembola), mites (Arachnida, Acari), earthworms (Oligochaeta, Crassiclitellata) and additional soil macrofauna. All specimens were collected from typical Argiudol soils located in three distinct agricultural systems characterised by varying levels of land-use intensity. To our knowledge, no other dataset exists providing this information for the Argentinian Pampas.

## Introduction

Soil-dwelling organisms are commonly classified by body size, using body width as the distinguishing morphological trait (Swift et al. 1979). These organisms fall into three categories: microfauna (width < 200 *μm*), mesofauna (width < 2 mm) and macrofauna (width > 2 mm). These categories are essential for understanding the roles different organisms play in soil ecosystems. All these organisms inhabit the spaces, or interstices, formed between surface litter (Wallwork 1958, Ritz and van der Putten 2012) and the porous network within the soil (Kampichler 1995, Lavelle 2012).

Within the mesofauna, mites (Arachnida, Acari) and springtails (Entognatha, Collembola) are the most abundant and diverse edaphic microarthropods, although, due to their body weights, they do not represent an important component of total edaphic metabolism (Hale et al. 2004). That being said, in the soil (Brussaard 2012), they are key actors in the functioning of the ecosystem, since they participate in the carbon and nutrient cycle through the consumption of organic matter, the transport of propagules, the control of microflora populations and of the microfauna and are the food resource for other edaphic organisms (Butcher and Snider 1971, Wurst et al. 2012).

Earthworms (Oligochaeta, Crassiclitellata) stand out within the macrofauna, since their presence contributes to the formation and maintenance of the physical structure of the soil, promoting aeration and permeability, which in turn provides optimal conditions for plant growth and the circulation of air, water and nutrients in the soil. In addition, due to their feeding mechanism, earthworms take the organic matter that accumulates in the soil, engulf it and deposit it as faecal pellets that are colonised by microorganisms, thus contributing to the humification processes and the release of nutrients (Rosswall et al. 1977, Paoletti 1999, Phillips et al. 2021).

The macrofauna does not present a high taxa diversity, but it does encompass a wide range of taxonomic ranks, differing at the level of orders and it plays a large number of functions in the edaphic ecosystem, such as herbivory, litter fractionation, control of populations by predators, transport of phoretic organisms and propagules of microorganisms and the formation of pores and habitats in the soil (Burges and Raw 1967, Lavelle and Allister 2001).

The taxonomic identification of the species that make up the community that inhabits the intricate network of pores and interstices of the soil is complex and, due to the great taxonomic diversity, its taxonomy is in constant revision and, furthermore, this identification becomes more difficult as the body size of the organisms decrease (Briones 2014).

All organisms respond to environmental pressures with individual changes in morphological, physiological, phenological or behavioural traits. The pressures that modify the characteristics of the environment are also reflected as changes in the population structure of the taxa under study (Sechi et al. 2017, Mittelbach and McGill 2019). Therefore, the effect of the interactions of organisms with their environment is reflected in the population variations and in the variations of the traits that can be used as indicators of ecological processes on a community level (Petchey and Belgrano 2010, Brussaard 2012).

Considering the above, the understanding of cryptic soil communities at the local level becomes necessary and it can be addressed through the use of individual traits without considering their identification to the species level. This would make it possible to understand the processes that occur in ecological communities and improve the analysis capacity of cryptic communities (Le Guillarme et al. 2023). The magnitude of the changes that occur in the edaphic fauna community could have a significant impact on the ecological and biogeochemical processes in the soil and, in turn, the ecosystem services they provide.

The edaphic fauna is sensitive to the disturbances that occur to the soil, because human activities alter the habitat and the source of the resources that these organisms use (Lavelle et al. 2006). For example, the pulses derived from the application of fertilisers and pesticides can alter the inputs and outputs of organic matter and nutrients; or when the soil is exposed to environmental factors during the fallow period, this can alter the conditions of the porous microclimate when the vegetation cover is not present; or in livestock systems in which soil compaction affects the physical structure, distribution and pore size distribution.

Variations in body size in ecological communities due to changes in the environment are analysed using the size spectrum (Pey et al. 2014), using the distribution of body weights and its relation to density (Turnbull et al. 2014). Analysing their relative abundance allows the description of the importance of different taxa in the community and can be related to functional redundancy and linked to ecosystem functioning (Briones 2014). Changes in the distribution of body weights in a community reflect variations in the environment or in the network of biological interactions (White et al. 2007, Pey et al. 2014). In turn, both relative abundance and body size distribution are closely related to the metabolism and the flow of energy that crosses the nodes in the network of interactions in the community (Potapov et al. 2019) of the soil system.

As described above, the changes in the size spectrum and in the biomass are linked to the response of the community to environmental pressures (Sechi et al. 2017), with the structure and dynamics of the communities (Jonsson et al. 2005) and with the functioning of the ecosystem (Peters 1999, Lavelle 2012) and they can show the effects of disturbance intensity on the soil ecosystem.

In this work, we present the dataset from GBIF data of Velazco et al. (2023) and the location of taxa of springtails (Entognatha, Colembolla), mites (Aracnida, Acari), earthworms (Oligochaeta, Crassiclitellata) and other macrofauna that occur in typical Argiudol soils under three different use systems, located in the Rolling Pampas Region in Argentina. This dataset contains the individual measurements of over 7000 individuals of the main morphological traits of each of the mentioned taxa: body length, body width and estimated body weight for each organism.

## Project description

### Title

Soil Biodiversity 2023: Size Spectra of the edaphic fauna of Argiudol soils typical of the Rolling Pampa Region, Argentina.

The project focuses on the characterisation of edaphic fauna on Argiudol soils of the Rolling Pampas, one of the most fertile and extensive agricultural plains in the world, under three intensities of human impact. By measuring the individuals found over a two year sampling period and calculating their biomass, we strive to estimate energy flux through different parts of the edaphic fauna and to estimate community stability. In this work, we present the complete dataset collected for the project. To the best of our knowledge, there is no other dataset for the Rolling Pampas that shows the spectrum of sizes and biomass of edaphic fauna for the different taxa found.

In this document, we present the list of taxa of springtails (Entognatha, Colembolla), mites (Aracnida, Acari), earthworms (Oligochaeta, Crassiclitellata) and other macrofauna that occur in typical Argiudol soils under three systems with different anthropogenic impact, located in the Argentinian Rolling Pampas Region. This list has individual measurements of the main morphological traits of each of the mentioned taxa, such as measurements of body length, body width and estimated body weight for each organism.

### Personnel

Victor Nicolás Velazco, Rosana V Sandler, Cynthia Sanabria, Carlos E Coviella, Lilliana B Falco, Leonardo A Saravia, Gabriel Tolosa, Anabela Plos

### Study area description

Samples were collected from fields located in the districts of Chivilcoy and Navarro in the Province of Buenos Aires, Argentina. The sampling sites were fields with three different intensities of land use: 1) Naturalised grasslands (N): abandoned grasslands without significant direct anthropogenic influence for at least 50 years, whose predominant vegetation is *Festucapratensis*, *Stipa* sp., *Cirsiumvulgare* and *Solanumlaucophylumm*; 2) Mixed livestock system (G): fields under continuous grazing with high animal load for 25 years, with a change towards forage production (bales of oats, corn and sorghum) for fattening two years prior to starting the study and 3) Agricultural system (A): fields under continuous intensive agriculture for 50 years and under no-tillage for the 18 years prior to the start of samplings.

### Design description

For each land use system, three different sites in separate fields were selected as replicates. In each replica, three sampling points were randomly located and then georeferenced to return to the same site on each sampling date.

### Funding

This project has been partially funded by a Doctoral Scholarship to Víctor Nicolás Velazco from the Concejo Nacional de Investigaciones Científicas (CONICET-Argentina), by the research programme in Terrestrial Ecology of the Universidad Nacional de Luján, with the support of the Instituto de Ecología y Desarrollo Sustentable (INEDES-UNLu-CONICET) and by Universidad Nacional de Lujan. There is also logistical support from the GBIF Argentina node, which is in charge of standards control, review and hosting of data and metadata.

## Sampling methods

### Study extent

The samples were taken from fields located in the districts of Chivilcoy and Navarro in Buenos Aires Province, Argentina.

The sampling sites were fields with three different intensities of land use: 1) Naturalised grasslands (N): abandoned grasslands without significant direct anthropic influence for at least 50 years, whose predominant vegetation is *Festucapratensis*, *Stipa* sp., *Cirsiumvulgare* and *Solanumlaucophylumm*; 2) Mixed livestock system (G): fields under continuous grazing with high animal load for 25 years, with a change towards forage production (bales of oats, corn and sorghum) two years prior to starting the study and 3) Agricultural system (A): fields under continuous intensive agriculture for 50 years and under no-tillage for the 18 years prior to the start of the samplings.

### Sampling description

The samplings were carried out once a season for 2 years. Soil subsamples with cores of 5 cm in diameter and 10 cm deep were taken at each sampling point. In order to obtain only the organisms living within the soil, the surface layer was gently brushed away before the soil samples were taken. Subsequently, the sample was homogenised and taken to the laboratory for the extraction of edaphic microarthropods using the flotation technique. In addition, at each sampling point, a 25 x 25 x 25 cm monolith was taken for the manual extraction of earthworms and other macrofauna organisms. The collected organisms were stored in 70% alcohol until their identification under a binocular microscope (Vargas and Recamier 2007, Moreira et al. 2012, Newton and Proctor 2013, Moretti et al. 2017).

### Step description

The edaphic microarthropods were extracted using the flotation technique, for which the homogenised sample was disaggregated and placed under water flow so that they pass through sieves with a 4 mm and 2 mm mesh opening, the soil that passed through the meshes was mixed in 2:1 ratio with a 1.2% magnesium sulphate solution.

The solution is allowed to settle for a few minutes until the mineral fraction of the soil settles and the supernatant in which the arthropods float is collected with a 98 um diameter sieve and stored in 70% alcohol until observation.

The collected supernatant was observed using a Leica S8P0 binocular microscope and, with the help of fine brushes and thin needles, the microarthropods were extracted and stored in 70% alcohol until their identification.

The identification of mites, springtails and worms and other fauna was carried out using taxonomic keys. After the identification, the body weights of the edaphic organisms were estimated, all of them expressed in micrograms of dry weight. The earthworms, after their identification, were weighed to determine the fresh weight, then they were dried under vacuum at 60 ºC and the dry weight factor of 0.15 on average was obtained (Rosswall et al. 1977)

The other organisms were measured one by one through photographs taken with a Leica S8P0 microscope with a built-in digital camera and whose rasters include a measurement scale depending on the configuration of the optical system at the time of capture.

Once the images were obtained, the ImageJ tool was used and the measurements of the body length and width of each of the individuals in micrometres were obtained.

Following this, several published linear equations relating body length and width were used to estimate the body weight of the organisms.

The length-width equations are general, but vary by taxonomic (Caruso and Migliorini 2009) group and also by the general shape that may exist within the taxonomic group. A total of 8662 specimens were measured individually.

## Geographic coverage

### Description

The Argentine pampa is a wide plain with more than 54 million hectares. Phytogeographically, it is located in the Neotropical Region, Chaqueño domain, Eastern district of the Pampean province and, therefore, the dominant vegetation is the steppe or pseudo-steppe of grasses (Cabrera 1976, Oyarzabal et al. 2018). The climate is temperate with 1100 mm of annual rainfall and an annual mean temperature of 17ºC. It has relatively high humidity throughout the year, periodically interrupted by droughts derived from El Niño and La Niña. The so-called Rolling Pampas is the most fertile and productive zone in the region, where more than 80% of the land is dedicated to the production of agricultural crops. The soils of the Pampas have relatively few limitations for crop production and are suitable for livestock. They are deep, well-drained soils, do not offer limitations for root growth and have a good organic matter content (Cabrera and Willink 1973).

The fields (Table 1) where all the samples were taken are located in the districts of Chivilcoy (60 m a.s.l. Lat: 35° 8'1.85"S Long: 59°44'41.37" W and Lat: 34°51'48.47" S Long: 60°13'10.51" W) and Navarro (43 m a.s.l. Lat: 34°49'12.72" S Long: 59°10'14.00" W) in the Province of Buenos Aires, Argentina. The fields with agricultural use are located within a radius of no more than 5 km from each other, the mixed fields that implement livestock and pasture cultivation are within a radius of less than 7 km and two of the three pastures are contiguous while the third is about 37 km distant. These distances in the Humid Pampa are practically irrelevant in terms of climate or elevation, the soils in all the sampled sites corresponding to typical Argiudols (Natural Resources Conservation Service et al. 2010) of the Henry Bell and Lobos series (CIRN 2022).

### Coordinates

-35.14 and -34.82 Latitude; -60.22 and -59.17 Longitude.

## Taxonomic coverage

### Description

The edaphic fauna organisms were classified into different taxonomic categories (Table 2). The identification of organisms stored in 70% alcohol was carried out with the support of taxonomic keys.

The mites were identified up to superfamilies (Burges and Raw 1967, Balogh and Balogh 1972, Evans and Till 1979, Balogh and Balogh 1988, Dindal 1990, Krantz and Walter 2009, Momo and Falco 2009), the springtails were identified up to the family level (Momo and Falco 2009, Claps et al. 2020, Janssens 2023), the earthworms, down to species (Satchell 1983, Reynolds 1996, de Michis and Moreno 1999) and the macrofauna was identified in different taxonomic ranks, whether they are classes, orders or families (Dindal 1990, Klimaszewiski and Watt 1997, Choate 1999, Zhang 2011, Vargas et al. 2014, Claps et al. 2020).

## Traits coverage

All the organisms of the edaphic fauna extracted by the sifting and flotation technique (Vargas and Recamier 2007) were processed; in total, for each system of use, 3530 - 3111 - 2021 animals were processed for the agricultural (A), livestock (G) and grassland (N) systems, respectively.

The organisms were taxonomically identified and then these organisms were characterised by their morphometric features. The morphometric traits measured were body length and body width, which allow the estimation of the body weight of each organism through the use of previously documented linear regression equations (Ganihar 1997, Newton and Proctor 2013).

**Figure 1.**
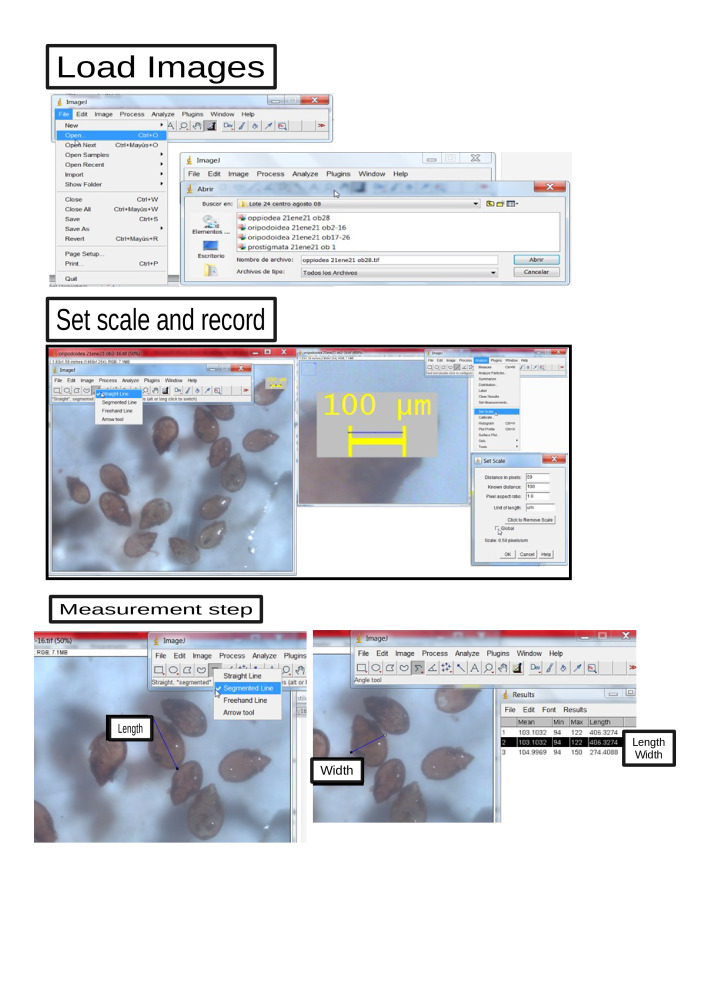
Graphic summary of the steps followed to obtain the measurements of the morphological traits, that is, the length and width of the body. Step one: upload the images to ImageJ. Step two: Configure the measurement tool through the relationship of the measurement scale and the length of pixels that it represents. Step three: take measurements of the lengths of interest.

Photographs of each member of the edaphic biota (see Fig. 1) stored in 70% alcohol were taken with a Leica stereoscope (S8AP0) with a camera included (Leica DFC 295) and with an integrated reference scale (Leica Application Suite V4.4). This allows micrometer precision to be obtained through the use of 40x eyepieces and a variable objective with a maximum magnification of up to 8x, which allowed working with magnifications of up to 320x.

To obtain the length measurements of the body length and width, each image was processed using the ImageJ software (Gonzales 2018, Rasband 2018), a programme for the processing of scientific images that allows measuring lengths in the images from a reference scale; each measurement obtained was recorded in this database.

**Figure 2.**
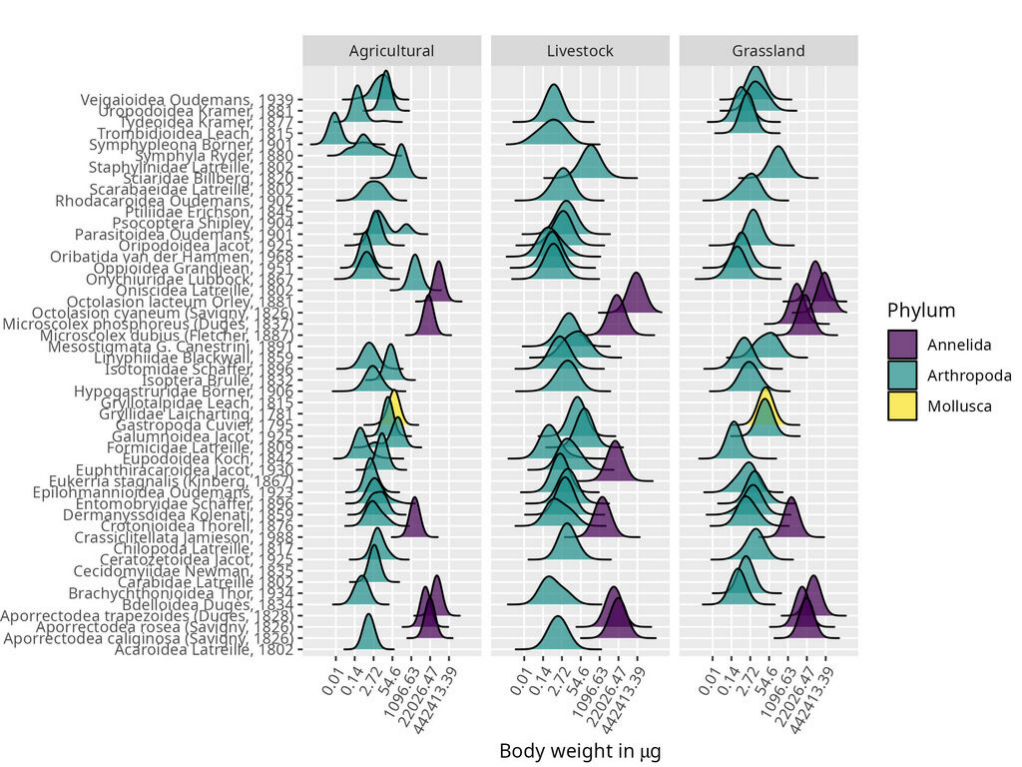
Density distribution of body weight in micrograms of dry weight of the taxa that make up the edaphic fauna community in the different land-use systems. Horizontal axis: body weight in micrograms on a logarithmic scale. Vertical axis: taxa by their scientific name. Legend: the colours refer to the phylum to which the different taxa belong.

Body weight estimates were made by using morphometric linear equations (Table 3) that relate the body lengths to the length and width of the edaphic fauna. These equations are taken from the scientific literature (Tanaka 1970, Lebrum 1971a, Lebrum 1971b, Petersen 1975, Rosswall et al. 1977, Hawkins et al. 1997, Hale et al. 2004, Greiner et al. 2010, Coulis and Joly 2017) and, in Fig. 2, the distribution of body weight of the different taxa involved is observed, which is the size spectrum of the fauna that inhabits the soil in the different management systems.

### Data coverage of traits

The dataset is then left with values of the following morphological traits: the body length and width in micrometres of the edaphic fauna, with the exception of earthworms and the body weight in micrograms of dry weight of each organism of the edaphic fauna found in the different sampling events.

## Temporal coverage

**Data range:** 2008-8-15 – 2010-12-15.

### Notes

The sampling design covered seasonal variability with bimonthly sampling over two years.

## Collection data

### Collection name

Size Spectra of the Edaphic Fauna from Rolling Pampas

### Parent collection identifier

Not applicable

### Specimen preservation method

Alcohol

## Usage licence

### Usage licence

Open Data Commons Attribution License

### IP rights notes

This work is licensed under a Creative Commons Attribution (CC-BY 4.0) License.

## Data resources

### Data package title

Size Spectra of the edaphic fauna of typical Argiudol soils of the Rolling Pampas Region, Argentina.

### Resource link

https://doi.org/10.15468/cmp3ma

### Alternative identifiers

https://www.gbif.org/dataset/6c685c4f-021a-40e2-a8a0-ac0ffc84c215

### Number of data sets

2

### Data set 1.

#### Data set name

Occurrence

#### Data format

Darwin Core

#### Download URL

https://www.gbif.org/occurrence/download?dataset_key=6c685c4f-021a-40e2-a8a0-ac0ffc84c215

#### Description

These datasets present the invertebrates of the edaphic fauna whose specimens belong to different taxa of Collembola, Entognatha (springtiails), Acari, Arachnida (mites), Crassiclitellata, Oligochaeta (earthworms) and other invertebrates of the edaphic fauna (Mollusca and Arthropoda) that are part of the macrofauna.

Each row records the presence of soil organisms and these were validated according to the Darwin Core Standard (DWC).

These soils are found in the Rolling Pampas Region, Argentina, one of the most extensive and fertile plains in the world. The data geographically references the sampling sites and also includes the date on which the samplings were taken.

**Data set 1.** 

Column label	Column description
occurrenceID	An unique identifier for the occurrence event.
institutionCode	The name in use by the institution.
collectionCode	The code identifying the collection.
catalogNumber	A unique identifier for the record within the dataset.
basisOfRecord	The specific nature of the data record: "Occurrence".
type	The nature or genre of the resource: "PhysicalObject".
datasetName	The name identifying the dataset.
habitat	A category for the habitat.
day	The integer day of the month on which the event occurred.
eventTime	The interval during which an event occurred.
otherCatalogNumbers	A list (concatenated and separated) of previous catalogue numbers.
higherGeography	A list (concatenated and separated) of geographic names less specific than the information captured in the country term.
continent	The name of the continent in which the event occurs.
country	The name of the country.
countryCode	The standard code for the country.
stateProvince	The name of the next smaller administrative region than country (province) in which the registry occurs.
county	The name of the smaller administrative region.
month	The integer month in which the event occurred.
year	The four-digit year in which the event occurred.
kingdom	The full scientific name of the kingdom in which the taxon is classified.
phylum	The full scientific name of the phylum in which the taxon is classified.
class	The full scientific name of the class in which the taxon is classified.
order	The full scientific name of the order in which the taxon is classified.
family	The scientific name of the family in which the taxon is classified.
genus	The genus part of the scientific name without authorship.
specificEpithet	The name of species epithet of the scientific name.
higherClassification	A list (concatenated and separated) of taxon names terminating at the rank immediately superior to the referenced taxon.
scientificName	The full scientific name or lowest level taxonomic rank that can be determined, with authorship and date information.
taxonRank	The taxonomic rank of the most specific name in the scientificName.
verbatimLatitude	The verbatim original latitude of the occurrence Location.
verbatimLongitude	The verbatim original longitude of the occurrence Location.
decimalLatitude	The geographic latitude, in decimal degrees.
decimalLongitude	The geographic longitude, in decimal degrees.
verbatimSRS	The ellipsoid, geodetic datum or spatial reference system (SRS), upon which coordinates given in verbatimLatitude and verbatimLongitude are based.
georeferencedBy	Names of people, who determined the georeference for the location occurrence.
recordedBy	Reference to the method used to determine the spatial coordinate names of people responsible for recording the original occurrence.
recordedByID	Globally unique identifier for the person responsible for recording the original occurrence.
samplingProtocol	Descriptions of the methods used during the event sampling.
sampleSizeValue	A numeric value for the size of a sample in a sampling event.
samplingEffort	The unit of measurement of the size of a sample in a sampling event. The amount of effort when sampling a event.
verbatimCoordinateSystem	The coordinate format for the verbatimLatitude and verbatimLongitude.
occurrenceRemarks	Notes about the occurrence.
eventDate	The date-time during which an event occurred.
sampleSizeUnit	The unit of measurement of the sample size of the sampling event.
georeferenceProtocol	A link to the reference on the methods used to determine the coordinates.

### Data set 2.

#### Data set name

Measurement: data set 2

#### Description

These datasets present the invertebrates of the edaphic fauna whose specimens belong to different taxa of Collembola, Entognatha (springtiails), Acari, Arachnida (mites), Crassiclitellata, Oligochaeta (earthworms) and other invertebrates of the edaphic fauna (Mollusca and Arthropoda) that are part of the macrofauna.

Each row records individual measurements of morphological traits of soil organisms that are extensions of the occurrence dataset described above and validated according to the Darwin Core Standard (DWC).

**Data set 2.** 

Column label	Column description
occurrenceID	A unique identifier taken from the occurrence dataset and linking it to the measurements of each occurrence.
measurementValue	The value of the measurement.
measurementUnit	The units associated with the measurementValue.
measurementType	The nature of the measurement.
measurementMethod	A description of the method used to determine the measurement.
measurementDeterminedBy	Names of people who determined the value of the measurement.
measurementDeterminedDate	Date range on which the measurement was taken.
measurementAccuracy	The description of the estimated error associated with the measurementValue.

## Figures and Tables

**Table 1.** Geographical location of the fields in which the samples were taken. Coordinates are in WGS84 sexagesimal degree systems.

System Use	Site field	Latitude / Longitude
Agricultural System	Casuarina	35°03'20.2''S, 59°41'18.5''W
	Molino	35°03'15.5''S, 59°41'09.9''W
	Manga	35°05'22.0''S, 59°38'70.9''W
Livestock System	L24	34°17'17.1''S, 59°10'31.3''W
	L25	34°49'25.0''S, 59°10'25.7''W
	L27	34°49'30.2''S, 59°10'16.1''W
Grassland	Romina	35°03'28.7''S, 59°41'03.6''W
	Festuca	35°03'31.6''S, 59°41'03.3''W
	Triángulo	34°51'05.0''S, 60°01'74.0''W

**Table 2.** Taxonomic coverage of edaphic fauna organisms.

**Phylum**	**Class**	**Order**	**Family**	**Scientific Name**	**Taxon Rank**
Mollusca	Gastropoda	Gastropoda		Gastropoda (Cuvier, 1795)	Order
Arthropoda	Symphyla			Symphyla (Ryder, 1880)	Class
	Pauropoda			Parasitoidea (Oudemans, 1901)	Class
	Malacostraca	Isopoda		Oniscidea (Latreille, 1802)	Suborder
	Insecta	Psocoptera		Psocoptera (Shipley, 1904)	Order
		Orthoptera	Gryllotalpidae	Gryllotalpidae (Leach, 1815)	Family
			Gryllidae	Gryllidae (Laicharting, 1781)	Family
		Hymenoptera	Formicidae	Formicidae (Latreille, 1809)	Family
		Diptera	Sciaridae	Sciaridae (Billberg, 1820)	Family
			Cecidomyiidae	Cecidomyiidae (Newman, 1835)	Family
		Coleoptera	Scarabaeidae	Scarabaeidae (Latreille, 1802)	Family
			Ptiliidae	Ptiliidae (Erichson, 1845)	Family
			Carabidae	Carabidae (Latreille 1802)	Family
				Staphylinidae (Latreille, 1802)	Superfamily
		Blattodea		Isoptera (Brullé, 1832)	Infraorder
	Entognatha	Symphypleona		Symphypleona (Börner, 1901)	Order
		Poduromorpha	Onychiuridae	Onychiuridae (Lubbock, 1867)	Family
				Hypogastruroidea (Börner, 1906)	Superfamily
		Entomobryomorpha	Isotomidae	Isotomidae (Schäffer, 1896)	Family
				Entomobryoidea (Schäffer, 1896)	Superfamily
	Chilopoda			Chilopoda (Latreille, 1817)	Class
	Arachnida	Trombidiformes		Tydeoidea (Kramer, 1877)	Superfamily
				Trombidioidea (Leach, 1815)	Superfamily
				Eupodoidea (Koch, 1842)	Superfamily
				Bdelloidea (Hudson, 1884)	Superfamily
		Oribatida		Oripodoidea (Jacot, 1925)	Superfamily
				Oribatida (van der Hammen, 1968)	Subclase
				Oppioidea (Grandjean, 1951)	Superfamily
				Galumnoidea (Jacot, 1925)	Superfamily
				Euphthiracaroidea (Jacot, 1930)	Superfamily
				Epilohmannioidea (Oudemans, 1923)	Superfamily
				Crotonioidea (Thorell, 1876)	Superfamily
				Ceratozetoidea (Jacot, 1925)	Superfamily
				Brachychthonioidea (Thor, 1934)	Superfamily
		Mesostigmata		Veigaioidea (Oudemans, 1939)	Superfamily
				Uropodoidea (Kramer, 1881)	Superfamily
				Rhodacaroidea (Oudemans, 1902)	Superfamily
				Parasitoidea (Oudemans, 1901)	Superfamily
				Mesostigmata (G. Canestrini, 1891)	Order
				Dermanyssoidea (Kolenati, 1859)	Superfamily
		Astigmata		Acaroidea (Latreille, 1802)	Superfamily
		Araneae	Linyphiidae	Linyphiidae (Blackwall, 1859)	Family
Annelida	Clitellata	Crassiclitellata	Lumbricidae	*Octolasionlacteum* (Örley, 1881)	Species
				*Octalacyumcyaneum* (Savigny, 1826)	Species
			Acanthodrilidae	*Microscolexphosphoreus* (Duges, 1837)	Species
				*Microscolexdubius* (Fletcher, 1887)	Species
			Ocnerodrilidae	*Eukerriastagnalis* (Kinberg, 1867)	Species
			Lumbricidae	*Apodorrectodeatrapezoides* (Duges, 1828)	Species
				*Apodorrectodearosea* (Savigny, 1826)	Species
				*Apodorrectodeacaliginosa* (Savigny, 1826)	Species
				Crassiclitellata (Jamieson, 1988)	Order

**Table 3.** Regression length-mass relationships with reference to the authors who estimated the regression equations and the body shape to which the different taxa fit. L = length of the body; l = width of the body; W = body weight; Log = base ten logarithm; ln = natural logarithm. The dry weight factor is inidicated only when neccesary for estimating dry weight.

Author	Body plan morphotype	Length-mass relationship equations	dry weight factor
Tanaka (1970)	Hipogastruridae	log W = 2,55 * log L + 0,99	
	Isotomidae	log W = 2,78 * log L + 0,71	
	Onychiuridae	log W = 2,75 * log L + 0,63	
	Entomobriidae	log W = 2,5 * log L + 0,83	
Petersen (1975)	Symphypleona	log W = log 39,6278 + 0,83 * log L	
Rosswall et al. (1977)	Trombidiformes	W = (0,00387 * L) ^3^	0.4
	Mesostigmata	W = 0,85 * (L ^2,09^ * l ^0,84^ * 10 ^-6,44^)	0.4
	Symphyla and Pauropoda	W=(1,20 + L) ^3^	0.2
Lebrum (1971a)	Achipteriforme oribatid’s	log W = 2,09 log L + 0,93 log l – 6,67	0.4
	Nothriforme oribatid’s	log W = 2,09 log L + 0,84 log l – 6,44	0.4
	Carabodiforme oribatid’s	log W = 1,62 log L + 1,40 log l – 6,56	0.4
	Acari	log W = 1,53 log L + 1,53 log l – 6,67	0.4
Hawkins et al. (1997)	Gastropoda	W = 0,172 L^1,688^	
Ganihar (1997)	Arannae	In W = - 3.2105 + L * 2.4681	
	Coleoptera adult	In W = - 3.2689 + L * 2.4625	
	Coleoptera larvae	In W = - 7,1392 + L * 0,8095	
	Diptera	In W = -3,4294 + L * 2,5943	
	Formicidae	In W = - 3,1415 + L * 2,3447	
	Insecta	In W = - 3,0710 + L* 2,2968	
	Isopoda	W = - 1,1167 + L * 0,4762	
	Pauropoda and Collembola	In W = - 1,8749 + L* 2,3002	
	Chilopoda	In W = - 6,7041 + L * 2,8420	
	Orthoptera	In W = - 3,5338 + L * 2,4619	
Coulis and Joly (2017)	Diplopoda Myriapoda	ln W = 2,38 * ln (L) – 2,77	0.45
